# Apoferritin Modified Magnetic Particles as Doxorubicin Carriers for Anticancer Drug Delivery

**DOI:** 10.3390/ijms140713391

**Published:** 2013-06-27

**Authors:** Iva Blazkova, Hoai Viet Nguyen, Simona Dostalova, Pavel Kopel, Maja Stanisavljevic, Marketa Vaculovicova, Marie Stiborova, Tomas Eckschlager, Rene Kizek, Vojtech Adam

**Affiliations:** 1 Department of Chemistry and Biochemistry, Faculty of Agronomy, Mendel University in Brno, Zemedelska 1, Brno CZ-613 00, Czech Republic; E-Mails: iva.blazkova@seznam.cz (I.B.); nguyenviethoai@hus.edu.vn (H.V.N.); esedinka@seznam.cz (S.D.); paulko@centrum.cz (P.K.); maja.stani85@gmail.com (M.S.); marketa.ryvolova@seznam.cz (M.V.); kizek@sci.muni.cz (R.K.); 2 Central European Institute of Technology, Brno University of Technology, Technicka 3058/10, Brno CZ-616 00, Czech Republic; 3 Department of Biochemistry, Faculty of Science, Charles University, Albertov 2030, Prague 2 CZ-128 40, Czech Republic; E-Mail: stiborov@yahoo.com; 4 Department of Paediatric Haematology and Oncology, 2nd Faculty of Medicine and University Hospital Motol, Charles University, V Uvalu 84, Prague 5 CZ-150 06, Czech Republic; E-Mail: tomas.eckschlager@fnmotol.cz

**Keywords:** cancer, nanomedicine, magnetic particles, doxorubicin, nanoparticles

## Abstract

Magnetic particle mediated transport in combination with nanomaterial based drug carrier has a great potential for targeted cancer therapy. In this study, doxorubicin encapsulation into the apoferritin and its conjugation with magnetic particles was investigated by capillary electrophoresis with laser-induced fluorescence detection (CE-LIF). The quantification of encapsulated doxorubicin was performed by fluorescence spectroscopy and compared to CE-LIF. Moreover, the significant enhancement of the doxorubicin signal was observed by addition of methanol into the sample solution.

## 1. Introduction

Magnetic particles can be the size of several nanometers to several micrometers and consist mainly of iron, nickel, cobalt and gadolinium [1–6]. Magnetic nanoparticles with appropriate surface coatings can be used for various biomedical purposes, such as drug delivery, hyperthermia, tissue repairing, cell and tissue targeting, transfection and magnetic resonance imaging [2,7–9].

Surface functionalization allows us to use nanoparticles as probes for molecular imaging [10]. The material employed for surface coating of the magnetic particles must be nontoxic and biocompatible and has to enable us a targeted delivery with localization in a required area [2,11]. Nanoparticles have a large surface area and provide a large number of functional groups for cross-linking to tumor-targeting ligands such as monoclonal antibodies, peptides, or small molecules for diagnostic imaging or delivery of therapeutic agents [12,13]. The linkage of the drug with magnetic nanoparticle has to be stable to prevent drug release during its transport [14]. Varied surface modifications can be used for the biomedical applications [11,15–17]. Polymers, poly(ethylene glycol) (PEG), *N*-(2-hydroxypropyl)methacrylamide (HPMA), and poly(lactide-*co*-glycolide) (PLGA) copolymers have been successfully utilized in clinical research [14]. Surface modifications can be also carried out using tetraethoxysilane (TEOS), triethoxysilane (TES) and 3-aminopropyltrimethoxysilane (APTMS) [6]. The transport to the vessel wall is essential for localizing therapy [18].

Superparamagnetic iron oxide could be used as an emerging therapeutic delivery system [19]. Anticancer drugs reversibly bound to magnetic fluids (ferrofluids) could be concentrated in tumors by magnetic fields that are arranged at the tumor surface outside of the organism [20]. Moreover, delivery by magnetic particles can be coupled to specialized nanocarriers such as lipid- [21] and/or protein-based carriers [22,23] enabling selective release of the drug in the site of the action. Such release may be performed by various mechanisms including photo- [24] or thermoiniciated [25] or pH triggered release [23,26]. In this study, magnetic particle-based targeted, apoferritin mediated and pH triggered transport of doxorubicin (DOX) was studied using capillary electrophoresis with laser-induced fluorescence detection.

## 2. Results and Discussion

**Figure 1:**
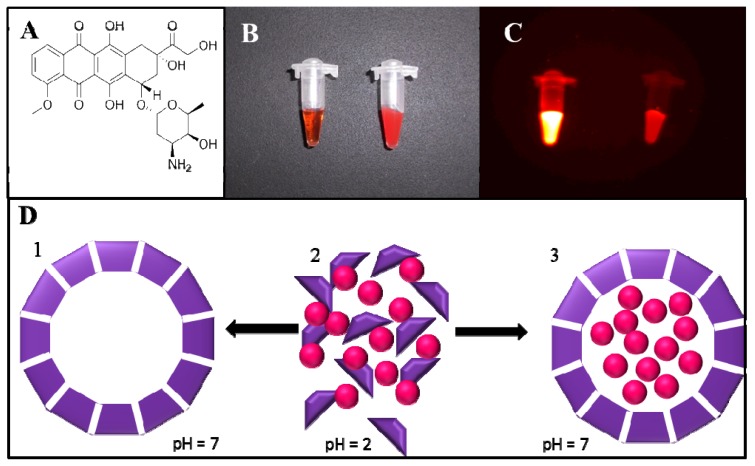
(**A**) Chemical structure of doxorubicin (DOX); (**B**) Photograph of solution of DOX (left) and apoferritin encapsulated doxorubicin (APODOX) (right) in the ambient light; (**C**) Fluorescence photograph of solution of DOX (left) and APODOX (right)— λ_ex_ = 480 nm, λ_em_ = 600 nm, exposition time 6 s, f_Stop_ 1.1, FOV 7.2; (**D**) Scheme of pH dependent disassembling and reassembling of apoferritin and encapsulation of DOX (1—schematic structure of assembled apoferritin at physiological conditions, 2—mixture of disassembled apoferritin units and DOX molecules at pH 2, 3) encapsulation of DOX molecules into the apoferritin cavity at increased pH and formation of APODOX).

Apoferritin is a protein composed of 24 polypeptide subunits, structurally arranged to create an internal cavity with size of 8 nm in diameter [27]. This cavity is naturally used for storage of iron ions; however, artificially it can be employed for carrying of any molecule of interest. Doxorubicin (DOX), an anthracycline antibiotic, which is due to its structure (Figure 1A) exhibiting an intrinsic fluorescence, belongs to such molecules. The encapsulation of DOX into the apoferritin cavity (formation of APODOX) is reflected in the fact that non-fluorescent apoferritin becomes fluorescent. Photograph of solution of DOX and APODOX in the ambient light is shown in Figure 1B and fluorescence photograph of solution of DOX and APODOX is shown in Figure 1C. The disassembling and reassembling mechanism based on pH of the environment is schematically illustrated in Figure 1D. The apoferritin structure is assembled at physiological conditions and by decreasing the pH to highly acidic region (pH 2) the protein is disassembled to its subunits. The mixture of apoferritin subunits and DOX molecules creates the basic solution for the encapsulation process. By increasing the pH of this solution the apoferritin structure is reassembled and DOX molecules are encapsulated in the cavity. This feature allows the application of apoferritin as a drug nanocarrier with specific low pH initiated release. For microencapsulation using the so-called “double emulsion” method, proteins in solution state may easily leak to the outer aqueous continuous phase, resulting in unacceptable low encapsulation efficiency [28–30]. Replacing the inner protein solution with solidified protein particles may substantially improve encapsulation efficiency, but protein particles still have the chance to contact with the outer aqueous continuous phase, leading to considerable loss of proteins. In general, higher encapsulation efficiency may be obtained by atomizing a protein-in-polymer suspension through a drying (or solidification) atmosphere prior to entering a collecting buffer [31].

### 2.1. Fluorimetric Characterization

**Figure 2:**
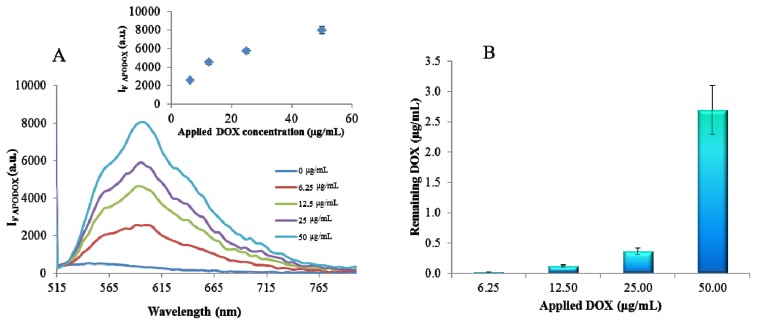
Fluorimetric characterization of APODOX. (**A**) Emission spectra (λ_ex_ = 480 nm) of APODOX prepared using various concentrations of DOX (0, 6.25, 12.5, 25, 50 μg/mL) and 1 mg/mL of apoferritin (Inset: Dependence of fluorescence intensities in the maximum (600 nm) on applied DOX concentration); (**B**) Dependence of total DOX concentration (free and encapsulated form) in the solution on concentration of applied DOX.

DOX exhibits the fluorescence with excitation maximum of 480 nm and emission maximum at 600 nm. Its encapsulation into the apoferritin cage does not influence the emission maximum and no shift is observed and the fluorescence intensity increased with the increasing concentration of free DOX used for encapsulation reaction (Figure 2A). However, the significant decrease in the intensity compared to the free DOX at the same concentration was detected. This decrease is caused by the incomplete encapsulation and by the removal of the free DOX using dialysis. According to the calibration curve of DOX with regression equation y = 1131.2ln(x) + 6870.8 the amount of encapsulated DOX was determined. It was observed that the encapsulation yield increased with the increased applied DOX concentration (Figure 2B).

### 2.2. CE Characterization

**Figure 3:**
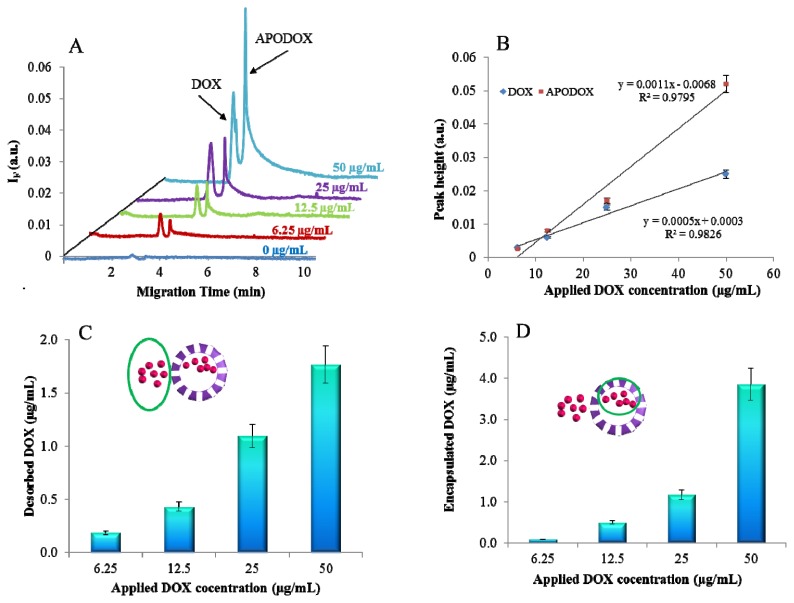
CE characterization of APODOX. (**A**) Typical electropherograms of APODOX solutions prepared by different concentrations (0, 6.25, 12.5, 25 and 50 μg/mL) of applied DOX; (**B**) Dependence of peak heights of DOX and APODOX peaks on the concentrations of applied DOX (0, 6.25, 12.5, 25, 50 μg/mL); (**C**) Dependence of the concentration of free DOX in the solution on the applied DOX concentration (0, 6.25, 12.5, 25 and 50 μg/mL); (**D**) Dependence of encapsulated DOX concentration on the applied DOX concentration (0, 6.25, 12.5, 25 and 50 μg/mL).

Capillary electrophoresis offers several benefits compared to stationary fluorimetric analysis. The most important of these is the ability to distinguish different forms of doxorubicin. As it is shown in Figure 3A, certain portion of free DOX was still remaining in the solution of the sample of APODOX even after the purification by dialysis. Therefore two peaks can be found in the electropherogram. The one with migration time of 2.9 min was identified as free DOX and the peak with migration time of 3.3 min belonged to the APODOX. At first, the hypothesis of inefficient dialysis purification was adopted. For this reason 3 times repeated 24 h long dialysis was performed and the fluorescence was acquired after each step. The fluorescence intensities of all three measurements were equal. Based on these results we came to the conclusion that some molecules of DOX were adsorbed on the surface of APODOX and therefore the dialysis purification was not capable of removing them. However, we assume that these molecules were desorbed from the APODOX surface in the CE probably due to the presence of the electric field and therefore a peak of free DOX can be detected in the electropherograms. The intensity of both peaks (DOX and APODOX) increased linearly with the increased concentration of applied DOX (Figure 3B). The lower slope of the DOX curve can be explained by gradual saturation of the APODOX surface by DOX molecules, which are subsequently desorbed.

Based on calibration curve for DOX with the regression equation y = 1.34x + 0.0013 the amount of DOX in both forms was quantified. The dependence of the amount of free DOX on the DOX amount applied to the encapsulation process is shown in Figure 3C. It follows from the results that the increasing applied amount led to the increased amount of desorbed DOX, however at the same time the amount of the encapsulated DOX (Figure 3D) increased nearly twice as much.

### 2.3. pH Triggered DOX Release

**Figure 4:**
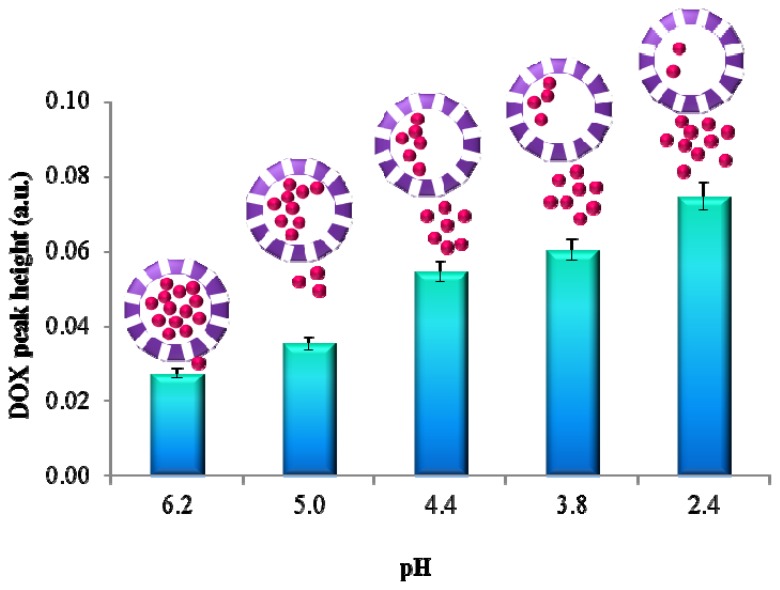
Dependence of fluorescence intensity of DOX peaks (obtained by CE-LIF, λ_ex_ = 480 nm, λ_em_ = 600 nm) released from APODOX by pH change. Insets: the scheme of DOX released from APODOX by decrease of pH.

It has been established that employment of separation technique such as CE-LIF is beneficial for APODOX investigation due to the ability to distinguish between encapsulated and desorbed DOX occurring in the solution. This can be utilized for monitoring of selective release of the drug. As noticed above lowering of pH to the highly acidic range leads to the release of the content of the apoferritin cavity. In the case of APODOX this was observed by the increasing of the intensity of the DOX peak in the APODOX electropherogram. The dependence of the peak intensity on pH is shown in Figure 4. This dependence is linear with regression equation y = 0.012x + 0.0146 and the coefficient of determination R^2^ = 0.9782. The results show that lowering the pH from 6.2 to 2.4 led to the increase of the desorbed DOX signal to the 272.9% of the original intensity. A schematic DOX release is illustrated by the schemes in the insets in Figure 4.

### 2.4. Influence of MeOH

**Figure 5:**
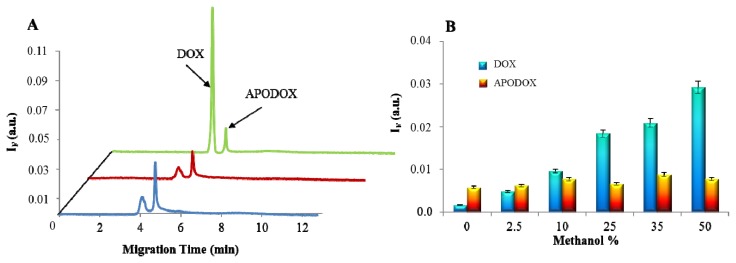
Methanol influence on fluorescent analysis of APODOX. (**A**) CE-LIF of APODOX (25 μg/mL) nondiluted (blue trace), diluted 1:1 by H_2_O (red trace) and diluted 1:1 by MeOH (green trace), for conditions see Figure 2; (**B**) Dependence of peak heights on MeOH percentage in the injected sample.

It was observed that the presence of MeOH in the sample improves the LIF detection during the CE analysis. It is shown in Figure 5A that, as expected, the dilution of APODOX by 1:1 by water led to the decrease in the fluorescence signal by 50%; however, dilution by MeOH caused an increase in the signal of desorbed DOX peak by 18-fold. Figure 5B shows the dependence of the height of DOX peak on the MeOH content in the analyte solution. It clearly follows from the results obtained that while the free (desorbed) DOX peak intensity increased linearly (R^2^ = 0.9778), the signal of APODOX peak remained unchanged.

### 2.5. Magnetic Particle Mediated APODOX Transport

**Figure 6:**
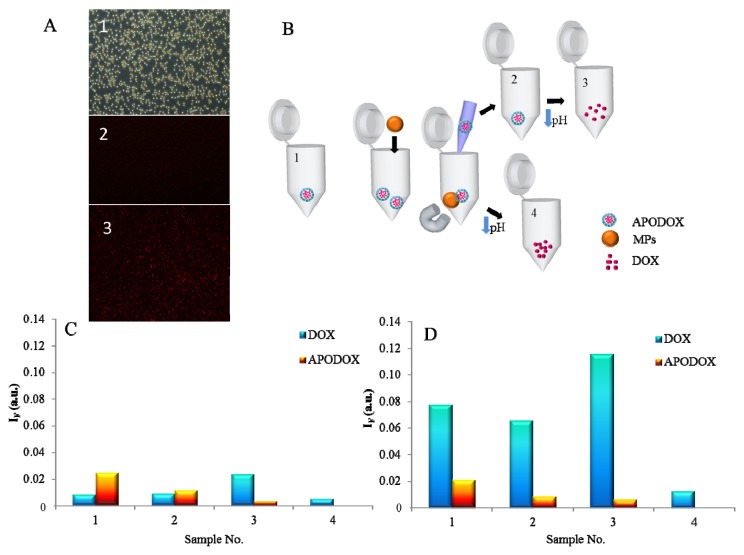
MPs-based transport of APODOX. (**A**) Micrographs of magnetic parties (1–unmodified MPs, 2–MPs modified by biotinylated apoferritin, 3–MPs modified by APODOX); (**B**) Scheme of the process–APODOX sample (No. 1) mixed with MPs, immobilized by magnetic field, unbound APODOX removed (No. 2), DOX released by pH decrease (No. 3), release of DOX from APODOX immobilized on MPs surface (No. 4); (**C**) Intensities of peaks (CE-LIF) in particular solution obtained during the procedure shown in panel (B); (**D**) Intensities of peaks (CE-LIF) in particular solution obtained during the procedure shown in panel (B) in 50% MeOH, for CE conditions see Figure 2.

First, magnetic particles and their conjugates with biotinylated APODOX were observed by fluorescence microscopy. The micrographs of the particles are shown in Figure 6A. The micrograph No. 1 shows unmodified particles under ambient light and the micrograph No. 2 shows the magnetic particles modified by the biotinylated apoferritin under illumination by the light with wavelength of 480 nm. As expected no fluorescence is observed, however the micrograph No. 3 shows a weak signal of particles modified by biotinylated APODOX.

The scheme of the procedure is shown in Figure 6B. Biotinylated APODOX (sample No. 1) was coupled to the streptavidin-functionalized magnetic particles and after application of an external magnetic field the unreacted solution was removed and analyzed by CE-LIF (Sample No. 2). Subsequently, the pH was decreased to release the DOX and the solution (Sample No. 3) was analyzed (CE-LIF). Finally, the magnetic particles with conjugated APODOX were resuspended in water and pH was decreased to release the DOX from the particles (Sample No.4). The peak intensities of all four solutions are summarized in Figure 6C.

## 3. Experimental Section

### 3.1. Apoferritin Encapsulated Doxorubicin (APODOX) Synthesis

Apoferritin solution 20 μL (50 mg/mL, equine spleen, Sigma-Aldrich) was diluted with 200 μL of ACS water. Doxorubicin (100 μL) was added and mixture was shaken. 1 M HCl (0.5 μL) was added and turbidity was observed. 15 min later 1M NaOH (0.5 μL) was added and turbidity disappeared. Solution was subsequently 2 h shaken on Vortex Genie2 (Scientific Industries Inc, Bohemia, NY, USA). Dialyses for 24 h were realized on membrane filter (0.025 μm VSWP, Millipore) against 2 L of water. Thus obtained solution was diluted with ACS water to final volume of 1 mL.

### 3.2. Magnetic Particle Mediated APODOX Transport

#### 3.2.1. Preparation of Biotinylated Apoferritin Filled with Doxorubicin

Biotinamidohexanoyl-6-aminohexanoic acid 100 μL (1 mg/mL) was added to the solution of APODOX prepared in Section 2.2. The mixture was shaken for 2 h on Vortex Genie2 and dialysis was accomplished as described in previous part.

#### 3.2.2. Modification of Streptavidin-Functionalized Particles with Biotinylated APODOX

Exactly 100 μL of streptavidin coated magnetic particles (Dynabeads M-270 Streptavidin, Life Technologies, Invitrogen, Prague, Czech Republic) in a clean vial were placed on a magnetic stand (Dynal MPC™-S, Life Technologies, Invitrogen, Prague, Czech Republic) and after the beads were immobilized on the vial by the magnetic field the storage solution was carefully removed by a pipette. The particles were subsequently washed (3×) by 100 μL of PBS buffer pH 7.4. After the last washing step was completed, 100 μL of biotinylated APODOX was added and the suspension was incubated for 30 min at 20 °C using Multi RS-60 Programmable rotator-mixer (Biosan, Riga, Latvia) (60 rpm, 90 °C). Subsequently the supernatant was removed using magnetic stand and the particles were washed 3× with PBS buffer pH 7.4. Finally, the washed particles were resuspended in the final volume (100 μL) of PBS buffer (pH 7.4).

### 3.3. Fluorescent Microscopy

Fluorescent microscopy was carried out using Mircoscope Olympus ix 71 (Olympus Czech Group Ltd., Pregue, Czech Republic) emploing an excitation wavelength of 480 nm and emission wavelength of 580 nm. The magnification was 200-times and the exposure time was 121 s.

### 3.4. Fluorimetric Analysis

Fluorescence spectra were acquired by multifunctional microplate reader Tecan Infinite 200 PRO (TECAN Group Ltd., Mannedorf, Switzerland). 480 nm was used as an excitation wavelength and the fluorescence scan in the range from 510 to 850 nm was measured every 5 nm. Each intensity value is an average of 5 measurements. The detector gain was set to 100. The sample (50 μL) was placed in transparent 96 well microplate with flat bottom by Nunc.

### 3.5. Capillary Electrophoresis with Laser-Induced Fluorescence Detection

Electrophoretic measurements were carried out using capillary electrophoresis system Beckman PACE/5000 with laser-induced fluorescence detection with excitation at 488 nm (CE-LIF). Uncoated fused silica capillary was used with total length of 47 cm and effective length of 40 cm. The internal diameter of the capillary was 75 μm. Tris-HCl buffer (50 mM, pH 8.2) was used as a background electrolyte and the separation was carried out using 20 kV with hydrodynamic injection for 20 s at 3.4 kPa.

## 4. Conclusions

The targeted therapy is a direction of current anticancer treatment and therefore numerous transporters are searched. Apoferritin enabling pH triggered content release in combination with magnetic particles is one of the systems providing required properties and behavior for this purpose. As shown in this work, doxorubicin can be effectively encapsulated into the apoferritin cavity and transported by magnetic field to the site of action.

Even though magnetic particles have been nowadays widely employed for target transport of a variety of cargos within the living organisms, their toxicity causing problems in terms of inflammation, formation of apoptotic bodies, generation of reactive oxygen species, and chromosome condensation has to be addressed. As shown in a number of studies, the toxicity can be influenced by a range of factors such as size, surface coating and/or surface charge. It is obvious that certain toxicity risks do exist and when magnetic particles are employed and a number of tests are required. However, we believe that the benefits of targeted delivery of extremely toxic cytostatic drugs by magnetic particles prevail over the disadvantages which are being moreover continuously eliminated by extensive research.

Similarly, the way of the most effective administration is widely investigated and so far the intravenous, subcutaneous and/or intratumoral fashion is generally accepted and performed even though each of these modes has both advantages and disadvantages.
